# Supplementary figures and images for: Genetic structure and relatedness of brown trout (Salmo trutta) populations in the drainage basin of the Ölfusá river, South-Western Iceland
Source: PeerJ. 2023 Sep 5;11:e15985. doi: 10.7717/peerj.15985 (PMC10487600; doi:10.7717/peerj.15985)

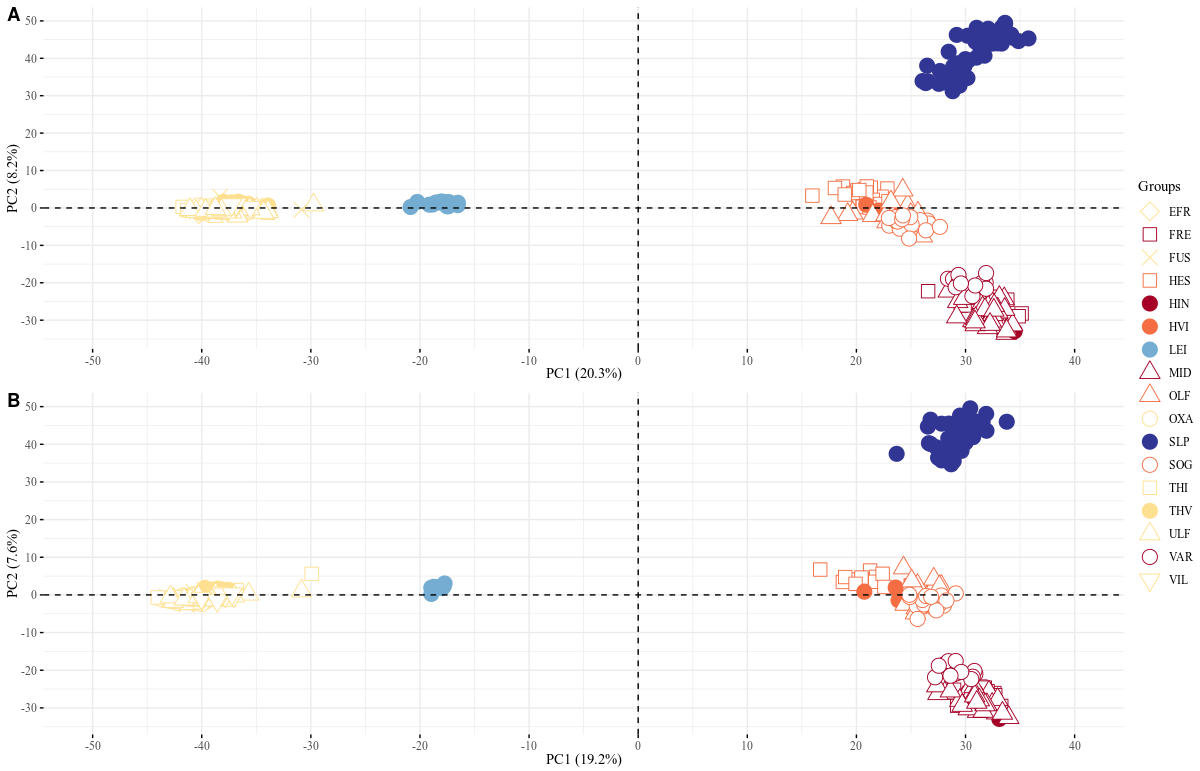

Supplement: Supplemental Information 1 — (A) PCA on de novo derived SNP’s. In total 2,597 putative neutral markers were ascertained after stringent filtering. (B) A dataset obtained from mapping to a reference genome, that resulted in 2,946 putative neutral markers using the same filtering criteria. Both show individuals and the two components that explained most of the data variation. See Table 1 for three letter code. [file peerj-11-15985-s001.png]

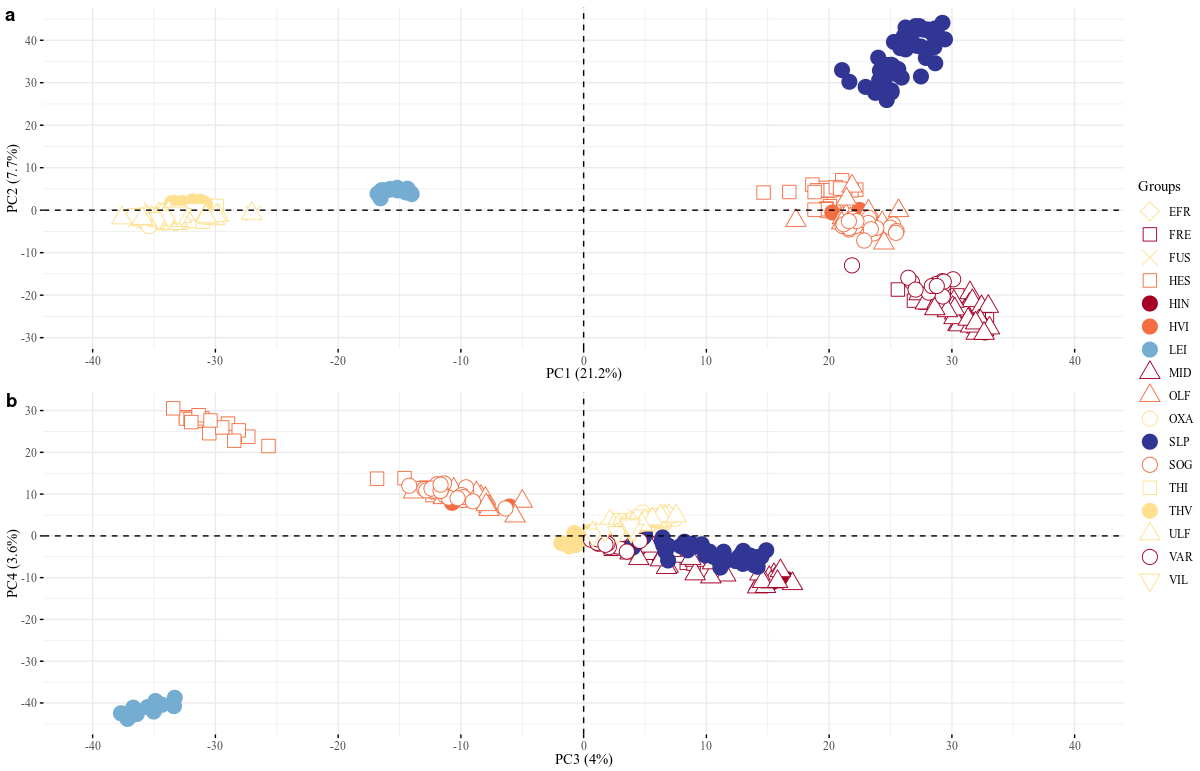

Supplement: Supplemental Information 2 — (A) PC1 = 21.2% and PC2 = 7.7%; (B) PC3 = 4% and PC4 = 3.6%. See Table 1 for three letter code. [file peerj-11-15985-s002.png]

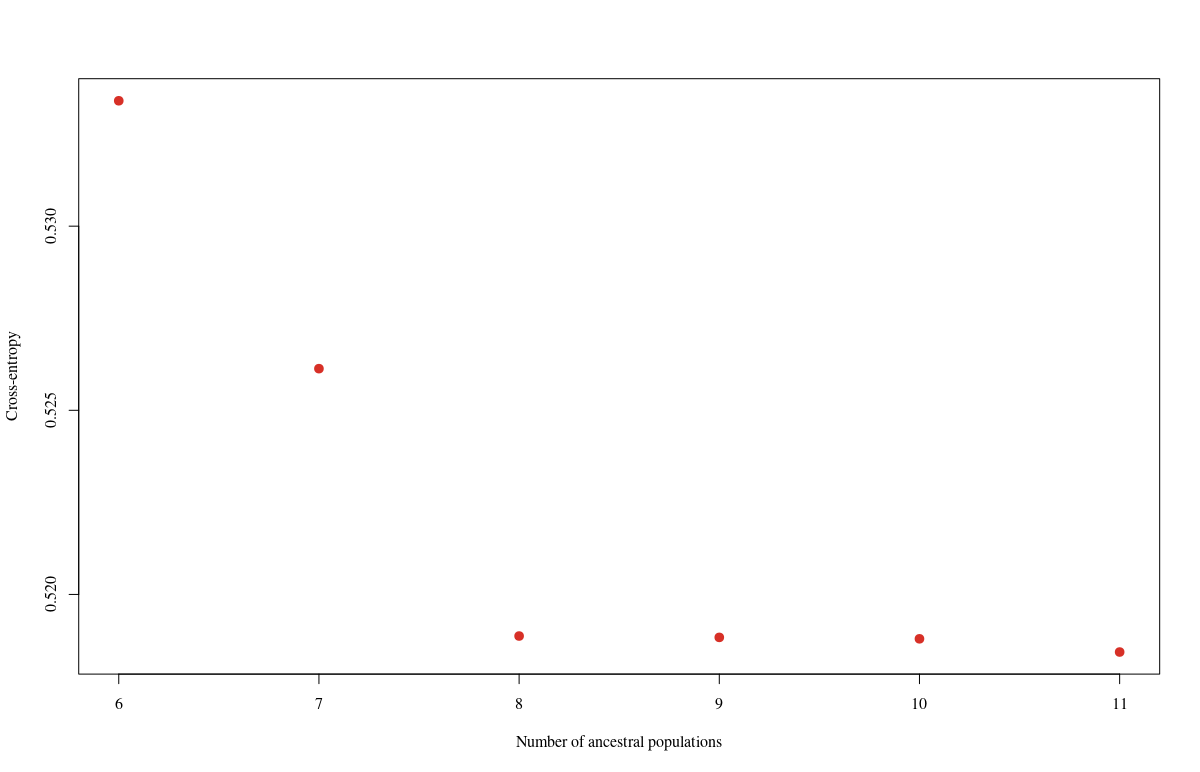

Supplement: Supplemental Information 3 — Estimated with the function snmf from the R package LEA (version 2.0). [file peerj-11-15985-s003.png]

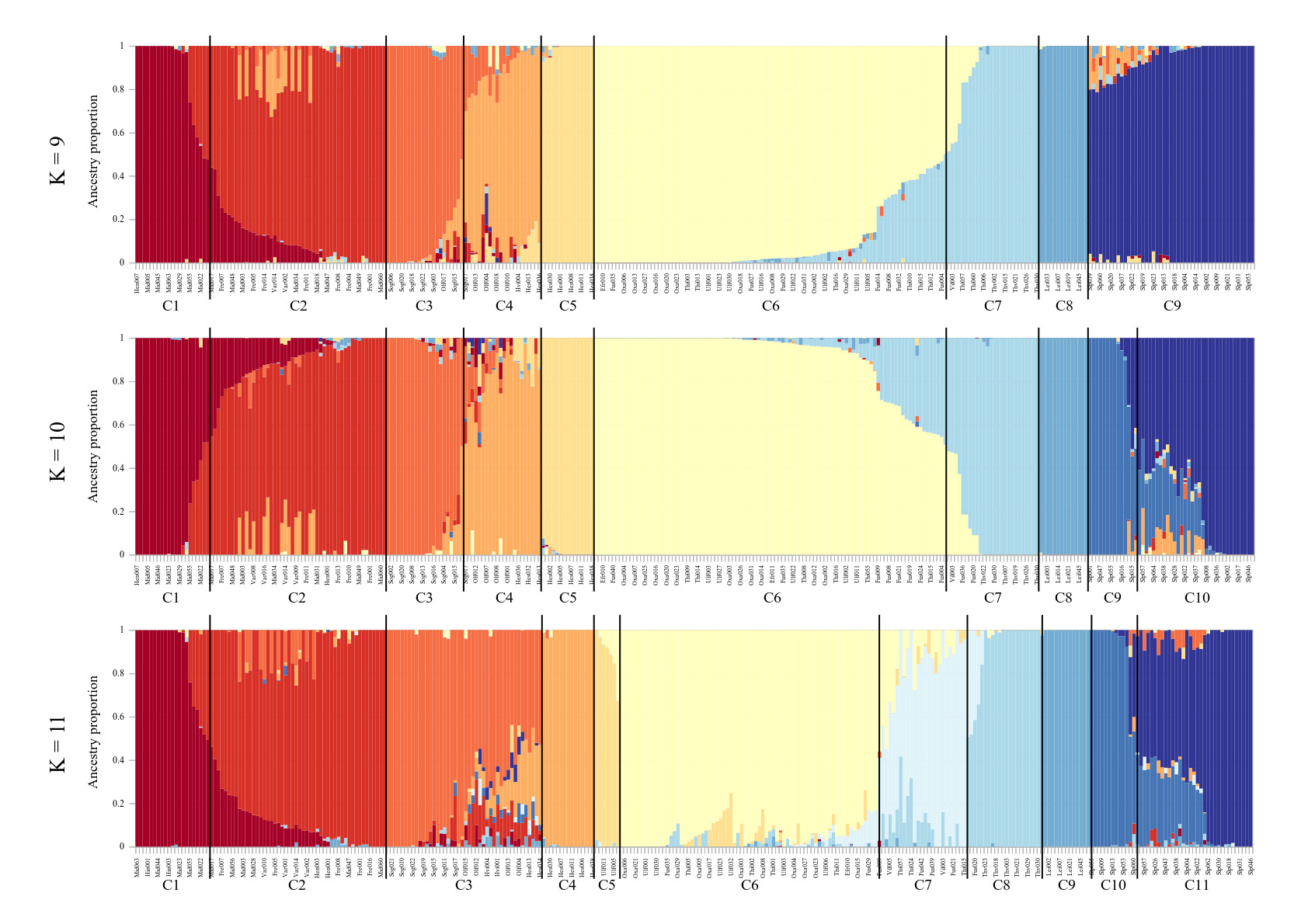

Supplement: Supplemental Information 4 — Note, not all the samples from each location grouped in the same cluster. Estimated with the function snmf from the R package LEA (version 2.0). [file peerj-11-15985-s004.png]
