# Supplementary material for: Genetic structure and relatedness of brown trout (Salmo trutta) populations in the drainage basin of the Ölfusá river, South-Western Iceland
Source: PeerJ. 2023 Sep 5;11:e15985. doi: 10.7717/peerj.15985 (PMC10487600; doi:10.7717/peerj.15985)
Supplement: Supplemental Information 5 — The eight BamHI-HF adapters were combined with 12 ApeK. adapters to generate 96 unique combinations. [file peerj-11-15985-s005.doc]

| **Restriction enzyme** | **Adapter name** | **Barcode** |
| --- | --- | --- |
| *Bam*HI-HF | Bam 04 | TAGAGC |
|  | Bam 05 | TATCTC |
|  | Bam 06 | AGGCAT |
|  | Bam 07 | TCCACT |
|  | Bam 08 | TTAGGT |
|  | Bam 09 | ACATAT |
|  | Bam 10 | TACGAT |
|  | Bam 11 | ACTGGC |
| *Ape*KI | Ape 02 | TTCTC |
|  | Ape 03 | TCGTT |
|  | Ape 09 | CTGTA |
|  | Ape 11 | ACCGT |
|  | Ape 13 | GCTTA |
|  | Ape 15 | GGTGT |
|  | Ape 17 | AGGAT |
|  | Ape 19 | ATTGA |
|  | Ape 21 | CATCT |
|  | Ape 23 | CCTAC |
|  | Ape 25 | GAGGA |
|  | Ape 27 | GGAAC |
