# Supplementary material for: Genetic structure and relatedness of brown trout (Salmo trutta) populations in the drainage basin of the Ölfusá river, South-Western Iceland
Source: PeerJ. 2023 Sep 5;11:e15985. doi: 10.7717/peerj.15985 (PMC10487600; doi:10.7717/peerj.15985)
Supplement: Supplemental Information 6 [file peerj-11-15985-s006.doc]

| **Stage** | **Program/script** | **Parameter** | **Description** | **Value** | **Number of loci retained** |
| --- | --- | --- | --- | --- | --- |
| Assembly of loci | process_radtags (Stacks) | -D | capture discarded reads to a file |  |  |
|  |  | -r | rescue barcodes and RAD-Tags |  |  |
|  |  | -t | truncate final read length to value (bp) | 115 |  |
|  |  | --renz_1 | restriction enzyme #1 | apeKI |  |
|  |  | --renz_2 | restriction enzyme #2 | bamHI |  |
|  |  | --inline_inline | barcode is inline with sequence |  |  |
|  |  | -i | input file type | gzfastq |  |
|  |  | -E | encoding of quality scores | phred33 |  |
|  |  | --clean | remove any read with an uncalled base |  |  |
|  |  | --adapter_1 | adapter sequence #1 | AGATCGGAAGAGCGTCGTGTAGGGAAAGAGTGT |  |
|  |  | --adapter_2 | adapter sequence #2 | AGATCGGAAGAGCGGTTCAGCAGGAATGCCGAG |  |
|  |  | --adapter_mm | mismatches allowed in the adapter sequence | 2 |  |
|  |  | --paired | files are paired |  |  |
|  |  | --quality | discard reads with low quality scores |  |  |
|  | ustacks (Stacks) | -M | mismatches allowed between stacks | 2 |  |
|  |  | -m | min. depth of coverage required to create a stack | 3 |  |
|  | cstacks (Stacks) | -n | mismatches allowed between loci | 2 |  |
|  | populations (Stacks) | -r | min. % of individuals in a pop. required to process a locus | 0.66 |  |
|  |  | --min_maf | min. minor allele freq. | 0.05 |  |
|  |  | --max_obs_het | max. observed heterozygosity | 0.6 | 44,343 |
| Removing paralogous sequences | paralog-finder | --minD | min. read ratio deviation | -4 |  |
|  |  | --maxD | max. read ratio deviation | +4 |  |
|  |  | --maxH | max. proportion of heterozygotes in a locus | 0.45 |  |
|  | populations (Stacks) | --write-single-snp | restrict data to only the first SNP per locus |  |  |
|  |  | -r | min. % of individuals in a pop. required to process a locus | 0.66 |  |
|  |  | --blacklist | loci blacklisted by paralog-finder were input to populations |  |  |
|  |  | --min_maf | min. minor allele frequency | 0.05 |  |
|  |  | --max_obs_het | max. observed heterozygosity | 0.6 | 20,532 |
| Data filtering | vcftools | --mac | min. minor allele count | 3 |  |
|  |  | --maf | min. minor allele freq. | 0.05 |  |
|  |  | --minDP | min. genotype freq. | 3 | 16,535 |
| Removing missing loci | vcftools | --exclude-positions | loci missing in > 40% of each population were removed |  | 4,105 |
| Removing loci out of HWE | filter_hwe_by_pop.pl | -h | min. cutoff for HWE p-value | 0.05 |  |
|  |  | -c | allowed % of all pop. that a locus can be below cutoff | 0.01 | 2,597 |
