# Supplementary material for: Genetic structure and relatedness of brown trout (Salmo trutta) populations in the drainage basin of the Ölfusá river, South-Western Iceland
Source: PeerJ. 2023 Sep 5;11:e15985. doi: 10.7717/peerj.15985 (PMC10487600; doi:10.7717/peerj.15985)
Supplement: Supplemental Information 7 — N20X: samples that were below the average coverage threshold of 20X; NKIN: individuals removed due to a high relatedness score; NMIS: individuals missing more than 60% of data. NDNA denotes samples used in most data analyses. N15X: number of individuals added back in after relaxing the coverage threshold to 15X in order to increase the number of samples for the effective population analysis and the detection of population bottleneck signals. Note that not all extra samples were used since the filters for missingness and kinship were re-applied. [file peerj-11-15985-s007.doc]

| 1. **Name** | 1. **Code** | 1. **NSEQ** | 1. **N20X** | 1. **NKIN** | 1. **NMIS** | 1. **NDNA** | 1. **N15X** |
| --- | --- | --- | --- | --- | --- | --- | --- |
| 1. Efra Sog | 1. EFR | 1. 15 | 1. -2 | 1. -8 | 1. 0 | 1. 5 | 1. 0 |
| 1. Hengladalsá *(Innstidalur)* | 1. HIN | 1. 6 | 1. 0 | 1. -3 | 1. 0 | 1. 3 | 1. 0 |
| 1. Hengladalsá *(Fremstidalur)* | 1. FRE | 1. 16 | 1. -1 | 1. 0 | 1. 0 | 1. 15 | 1. +1 |
| 1. Hengladalsá *(Miðdalur)* | 1. MID | 1. 70 | 1. -16 | 1. -14 | 1. 0 | 1. 40 | 1. +8 |
| 1. Hestvatn | 1. HES | 1. 38 | 1. -15 | 1. 0 | 1. -2 | 1. 21 | 1. +11 |
| 1. Hvítá | 1. HVI | 1. 7 | 1. -4 | 1. 0 | 1. 0 | 1. 3 | 1. +3 |
| 1. Leirvogsvatn *(local reference)* | 1. LEI | 1. 65 | 1. -47 | 1. -2 | 1. -2 | 1. 14 | 1. +11 |
| 1. Sog | 1. SOG | 1. 24 | 1. -1 | 1. -4 | 1. 0 | 1. 19 | 1. +1 |
| 1. Úlfljótsvatn | 1. ULF | 1. 32 | 1. -7 | 1. -4 | 1. 0 | 1. 21 | 1. +4 |
| 1. Varmá | 1. VAR | 1. 18 | 1. -4 | 1. 0 | 1. -1 | 1. 13 | 1. +2 |
| 1. Villingavatnsá | 1. VIL | 1. 11 | 1. 0 | 1. -7 | 1. 0 | 1. 4 | 1. 0 |
| 1. Þingvallavatn | 1. THI | 1. 60 | 1. -35 | 1. 0 | 1. 0 | 1. 25 | 1. +28 |
| 1. Þverá/upper Ölfusvatnsá | 1. THV | 1. 36 | 1. -3 | 1. -18 | 1. 0 | 1. 15 | 1. +3 |
| 1. Ölfusá | 1. OLF | 1. 18 | 1. -2 | 1. 0 | 1. 0 | 1. 16 | 1. 0 |
| 1. lower Ölfusvatnsá | 1. FUS | 1. 44 | 1. -7 | 1. -12 | 1. 0 | 1. 25 | 1. +6 |
| 1. Öxará | 1. OXA | 1. 31 | 1. 0 | 1. 0 | 1. 0 | 1. 31 | 1. 0 |
| 1. Loch Slapin *(reference group)* | 1. SLP | 1. 64 | 1. -13 | 1. -4 | 1. 0 | 1. 47 | 1. +1 |
