# Supplementary material for: Genetic structure and relatedness of brown trout (Salmo trutta) populations in the drainage basin of the Ölfusá river, South-Western Iceland
Source: PeerJ. 2023 Sep 5;11:e15985. doi: 10.7717/peerj.15985 (PMC10487600; doi:10.7717/peerj.15985)
Supplement: Supplemental Information 8 — N: sample size; HE: expected heterozygosity; HO: observed heterozygosity; π: nucleotide diversity. A star indicates pairs of HE/HO that showed significant differences (p < 0.05, Bartlett test). [file peerj-11-15985-s008.doc]

| **Code** | **N** | **H_E_[[1]](#footnote-2)** | **H_O_[[2]](#footnote-3)*** | **π (x 10^-4^)** |
| --- | --- | --- | --- | --- |
| EFR | 5 | 0.128* | 0.127* | 5.04 |
| FUS | 25 | 0.147 | 0.145 | 5.18 |
| OXA | 31 | 0.132 | 0.132 | 5.42 |
| THI | 25 | 0.148 | 0.140 | 4.30 |
| ULF | 21 | 0.147* | 0.147* | 5.39 |
| VIL | 4 | 0.144* | 0.143* | 5.64 |
| THV | 15 | 0.085 | 0.080 | 3.26 |
| HIN | 3 | 0.181* | 0.193* | 6.87 |
| MID | 40 | 0.252 | 0.239 | 9.37 |
| FRE | 15 | 0.252* | 0.250* | 10.05 |
| HES | 21 | 0.258 | 0.240 | 9.13 |
| HVI | 3 | 0.301* | 0.289* | 10.42 |
| SOG | 19 | 0.288* | 0.284* | 10.98 |
| OLF | 16 | 0.306* | 0.289* | 11.63 |
| VAR | 13 | 0.417* | 0.272* | 9.50 |
| LEI | 14 | 0.089 | 0.080 | 2.58 |
| SLP | 47 | 0.300 | 0.280 | 10.65 |

1. A star indicates pairs of H_E_/H_O_ that showed significant differences (p < 0.05, Bartlett test). [↑](#footnote-ref-2)
2. ^*^ [↑](#footnote-ref-3)
