# Supplementary material for: Genetic structure and relatedness of brown trout (Salmo trutta) populations in the drainage basin of the Ölfusá river, South-Western Iceland
Source: PeerJ. 2023 Sep 5;11:e15985. doi: 10.7717/peerj.15985 (PMC10487600; doi:10.7717/peerj.15985)
Supplement: Supplemental Information 10 — NE values obtained with a dataset without linkage disequilibrium (LD) correction (5,353 loci, column header before), and with a second dataset obtained after LD correction (3,505 loci, column header after). The function filter_ld from the R package radiator was used to make sure that only one SNP was present in each read, and that a SNP’s filter was applied based on Long Distance Linkage Disequilibrium. [file peerj-11-15985-s010.doc]

|  |  | **N_E_** | | | **CI 2.5%** | | | **CI 97.5%** | | |
| --- | --- | --- | --- | --- | --- | --- | --- | --- | --- | --- |
| **Code** | **N** | **before** | **after** | **diff (%)** | **before** | **after** | **diff (%)** | **before** | **after** | **diff (%)** |
| FUS | 23 | 14.9 | 14.4 | -3.36 | 8.5 | 7.9 | -7.06 | 30.9 | 31.2 | 0.97 |
| OXA | 31 | 56.5 | 72.1 | 27.61 | 42.1 | 50.8 | 20.67 | 82.9 | 117.9 | 42.22 |
| ULF | 24 | 21 | 18.8 | -10.48 | 13 | 10.9 | -16.15 | 40 | 39.9 | -0.25 |
| THV | 23 | 14.7 | 15 | 2.04 | 9.7 | 9.3 | -4.12 | 24.3 | 26.8 | 10.29 |
| MID | 38 | 14.3 | 14 | -2.10 | 9.5 | 9.1 | -4.21 | 22 | 21.9 | -0.45 |
| HES | 30 | 10.5 | 10.2 | -2.86 | 7.5 | 7.5 | 0.00 | 14.7 | 14.1 | -4.08 |
| SOG | 20 | 71.2 | 76.6 | 7.58 | 41.2 | 43.8 | 6.31 | 209.3 | 241.9 | 15.58 |
| LEI | 20 | 3.4 | 5.6 | 64.71 | 2.7 | 3.1 | 14.81 | 6.3 | 8.5 | 34.92 |
| SLP | 48 | 44.7 | 47.5 | 6.26 | 34.2 | 35.9 | 4.97 | 61.6 | 66.3 | 7.63 |
